# Detection of microplastics in bladder wash fluid obtained after cystoscopy in patients diagnosed with bladder cancer

**DOI:** 10.3389/fpubh.2026.1812477

**Published:** 2026-05-20

**Authors:** Burak Yılmaz, Miraç Ataman, Ayşegül Tuna

**Affiliations:** 1 Department of Urology, Osmaniye Research and Training Hospital, Osmaniye, Türkiye; 2 Department of Urology, Siirt Research and Training Hospital, Siirt, Türkiye; 3 Department of Infectious Diseases, Kırıkkale University, Kırıkkale, Türkiye

**Keywords:** bladder cancer, bladder wash fluid, environmental waste, microplastics, urine

## Abstract

**Purpose:**

To evaluate the possible association between bladder cancer and the concentration of microplastics detected in urine.

**Methods:**

This retrospective observational study examined 76 patients presenting to the Urology Outpatient Clinic between December 2024 and May 2025. The patient group consisted of 40 individuals diagnosed with bladder cancer based on pathological results, while the control group comprised 36 volunteers who underwent cystoscopy but had no pathological lesions. During cystoscopy, 5 mL of urine was collected from each participant in sterile glass tubes for microplastic analysis. Microplastic particles were identified and quantified in urine samples using stereomicroscopy. The demographic characteristics, microplastic concentrations and clinical data of patients diagnosed with bladder cancer were recorded.

**Results:**

Participants were aged between 41 and 82 years, with an average age of 63.04 ± 10.15 years. No significant difference in age distribution was observed between the groups. The mean body mass index was 28.31 ± 4.79 kg/m^2^. All patients in the cancer group were diagnosed with non-muscle-invasive urothelial carcinoma. The mean microplastic concentration was 2.82 ± 2.05 particles/ml in the control group and 2.53 ± 1.54 particles/ml in the bladder cancer group. There was no statistically significant difference in microplastic concentration between the two groups (*p* = 0.868). Within the bladder cancer group, microplastic concentration did not differ significantly according to sex, lamina propria invasion, tumour size, tumour grade, number of tumour foci, presence of carcinoma *in situ* or smoking history (all *p* > 0.05).

**Conclusion:**

The findings of this study suggest that, under the conditions and analytical limitations of the present study, no significant association was observed between urinary microplastic concentration and urothelial carcinoma of the bladder. Further clarification of the potential relationship between bladder cancer and urinary microplastic levels requires larger-scale studies.

## 1 Introduction

Plastics are used in almost every area of modern life. While single-use plastic products are recyclable, the fact that they persist in the environment for many years means their negative ecological impact is undeniable. In addition to the plastic items we use every day, industrial waste also constitutes a significant component of plastic pollution. Every year, tons of plastic waste are released into the environment. Microplastics (MPs) are plastic particles smaller than 5 mm. These particles typically originate from the degradation of larger plastic materials under environmental conditions. They are produced and used in various sectors, including cosmetics, cleaning products, textiles, bottles and packaging materials, biomedical applications, fishing equipment and the automotive industry (1, 2). Plastic waste decomposes into microplastics through the physical and chemical effects of sunlight, water and air in oceans, lakes and rivers, as well as in terrestrial environments (3). Polyester-based fabrics release microplastic particles when worn or washed, and sportswear and water sports clothing are major contributors to this. Some cosmetic products, such as facial scrubs and toothpaste, may contain microplastic structures. Fishing nets, lines and other equipment left in marine environments can also generate microplastics over time (4). Microplastics can persist in the environment for extended periods, negatively affecting aquatic ecosystems. Fish and other marine organisms can ingest these particles, allowing microplastics to enter the food chain and have an adverse impact on the health of all living organisms, particularly aquatic species (5). Microplastics can enter the human body through ingestion, dermal contact or inhalation (6). Numerous studies have reported the presence of microplastics in contaminated water sources, as well as in the tissues of organisms living in these environments (7). Similar findings have been reported in food products. Consuming water stored in plastic bottles or water that is contaminated by plastic increases the likelihood of human exposure to high levels of microplastics. Plastics are complex, heterogeneous materials made up of carbon-based polymers. They also contain various synthetic additives, many of which are highly toxic. These include carcinogenic and neurotoxic agents such as bisphenols, perfluoroalkyl substances, polyfluoroalkyl substances, and organophosphates. These chemicals are integral components of plastics and are responsible for many of the adverse effects of plastics on human health and the environment. Research is ongoing to investigate the mechanisms through which microplastics may affect human health and contribute to disease development. Studies have demonstrated that MPs can be absorbed through the stomach and intestines following oral ingestion (8). Microplastics have been shown to cause alterations in intestinal epithelial permeability, growth retardation, behavioural changes, reproductive system toxicity and oxidative stress (4, 6). Studies in rats exposed to microplastics have demonstrated neurotoxicity, hepatotoxicity, gastrointestinal toxicity, oxidative stress and metabolic disorders. Furthermore, microplastics may delay the healing of pre existing diseases or exacerbate their severity (9). MPs are thought to contribute to the development of cancers such as lung, breast, prostate and ovarian cancer by triggering uncontrolled cell proliferation (10). They have also been associated with early-onset colorectal cancer and have been suggested as a potential factor in chemotherapy resistance (11). Chronic inflammation, characterised by tissue damage, angiogenesis and fibrosis, is recognised as a critical mechanism in cancer development. Numerous studies support the hypothesis that microplastic accumulation may trigger inflammatory responses, disrupt the microbiome, and activate immune responses due to their physicochemical properties (12, 13). There is also evidence to suggest that MPs may alter the tumour microenvironment, impair immune surveillance, influence cancer progression and thereby promote tumour development and metastasis (14). Furthermore, recent environmental health studies have demonstrated an increase in environmental exposure to airborne and waterborne microplastics (15). In the aetiology of bladder cancer, factors such as working in the dye and chemical industries, exposure to heavy metals, chemical irritation of the urothelium, infections and chronic inflammation play important roles. During bladder cancer development, disruptions occur in mechanisms regulated by p53, including apoptosis. Overexpression of proto-oncogenes has also been reported to contribute to bladder carcinogenesis (16). Microplastics have been detected in human renal tissue, as well as in urine samples obtained from healthy individuals (17, 18). However, the relationship between bladder cancer and microplastics remains unclear. There is emerging molecular evidence to suggest that environmental factors may contribute to carcinogenesis by affecting inflammatory and immune-modulating pathways (19). Therefore, we aimed to investigate whether microplastics, which have harmful effects on living organisms through various mechanisms, may play a role in bladder cancer development, particularly in the bladder epithelium which is in continuous contact with urine.

## 2 Materials and methods

**Figure 1:**
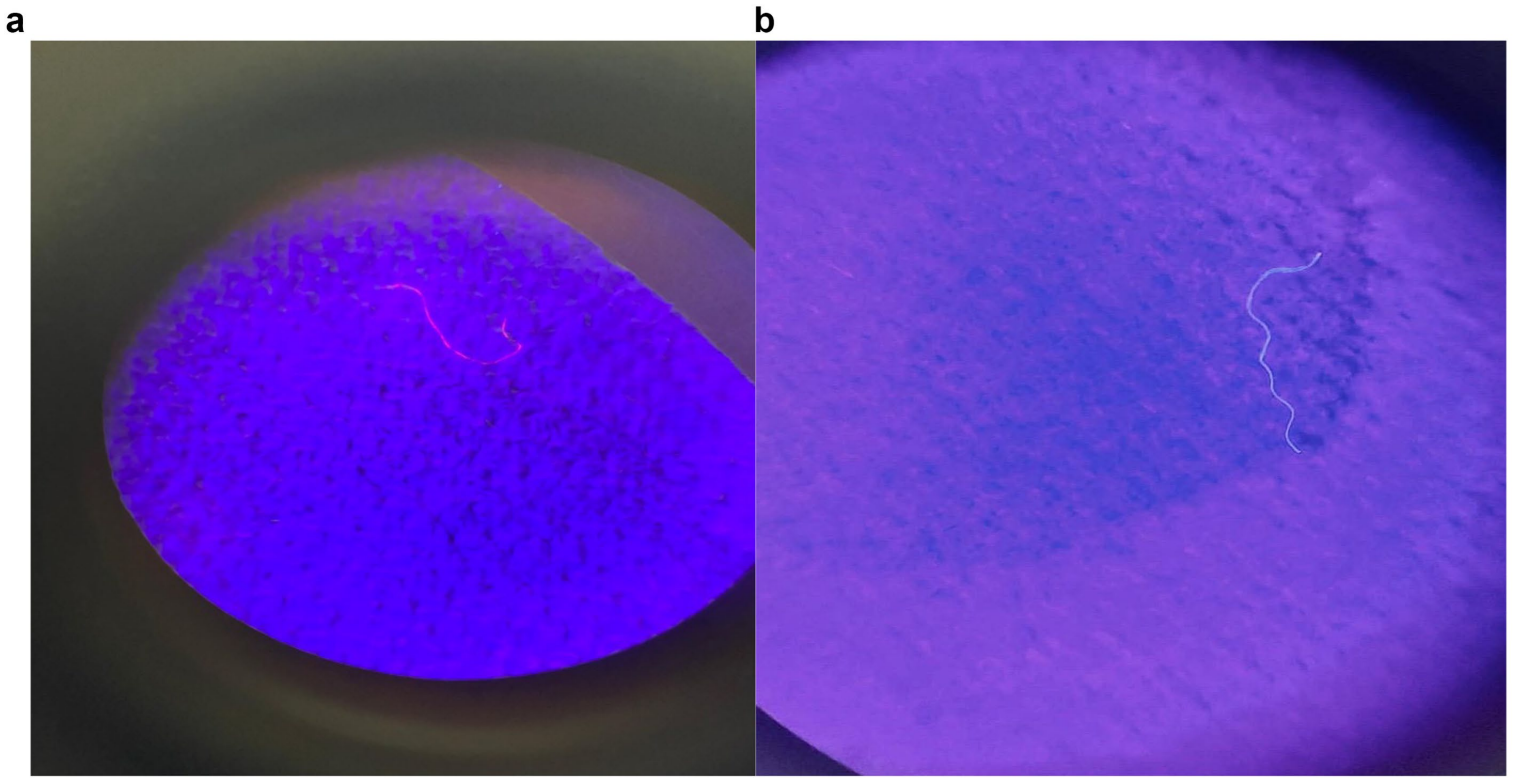
Visualization of fibrous microplastic particles. The images show characteristic thread-like structures stained with Nile Red and visualized via fluorescence microscopy (magnification ×250) **(a,b)**.

This retrospective observational study was conducted after ethical commitee approval was obtained from Osmaniye Korkut Ata University (Approval number: OKU.KKO.FR.0024 08/01/2025) and included 76 individuals who attended the Urology Outpatient Clinic at our hospital between December 2024 and May 2025. Participants were eligible if they had radiographic evidence of an intravesical tumour or haematuria, had not undergone any prior urological interventions and had a urinary tract infection ruled out. The patient group consisted of 40 individuals who met these criteria and were subsequently diagnosed with bladder cancer, while the control group comprised 36 patients who underwent cystoscopy due to microscopic hematuria, had no evidence of tumors or suspicious mucosal lesions on cystoscopic evaluation, and did not develop bladder cancer during follow-up. For the microplastic analysis, 5 mL of urine was collected from each participant during cystoscopy using a glass syringe, then transferred into sterile glass tubes. This approach is methodologically sound, as cystoscopy-guided sampling minimises external contamination and provides a specimen that is more anatomically representative than voided urine. Demographic and clinical data, including age, height, weight, body mass index (BMI), smoking status, tumour grade, presence of lamina propria invasion, tumour size, number of tumour foci and presence of carcinoma *in situ* (CIS), were obtained retrospectively from hospital records. For the laboratory analysis, chemical digestion was performed to remove the organic components present in the urine samples using Fenton’s reagent (30% hydrogen peroxide and 0.05 M iron sulphate). This procedure was carried out at 45 °C with continuous stirring on a magnetic mixer. After digestion, the samples were filtered through microfibre filter papers with a pore size of 1.2 μm using a stainless steel vacuum filtration system, and were then rinsed at least three times with distilled water. The filters were placed in sterile glass Petri dishes and incubated at 45 °C for 24 h. Following incubation, the filters were examined using an M80 stereomicroscope equipped with a Leica Flexacam C1 camera. Microplastic particles were visually identified and counted based on their morphological characteristics. The identity of the particles was confirmed using the hot needle test. Nile Red (CAS Number: 7385-67-3) staining was then applied, after which the fluorescence under blue light was evaluated to verify the composition of the microplastics (Figure 1). Combining hot needle testing with fluorescence-based visualisation strengthened the reliability of microplastic identification by providing complementary physical and optical confirmation. Although spectroscopic confirmation methods such as Fourier-transform infrared (FTIR) or Raman spectroscopy were unavailable, multiple complementary identification techniques were employed to minimise misclassification, including morphological evaluation, hot needle testing and Nile Red fluorescence staining. However, it should be noted that Nile Red staining may also bind to non-plastic organic materials, such as lipids, which could lead to false positives. To minimise the risk of external contamination, all distilled water used in the study was pre-filtered through a stainless steel filter. All glass and metal equipment that came into contact with the samples was cleaned with pre-filtered distilled water prior to use. Filter papers were inspected under the microscope prior to analysis and any contaminated filters were excluded. All laboratory procedures were conducted inside a biosafety cabinet while wearing cotton laboratory coats only. To prevent airborne contamination, glass and metal surfaces were covered with aluminium foil. Throughout the analysis, procedural blanks prepared with filtered distilled water were kept on the workbench and examined at the end of the study to monitor potential contamination. The data obtained from the study were analysed using IBM SPSS Statistics for Windows, Version 27.0 (IBM Corp., Armonk, NY, USA). Descriptive statistics were presented as frequency (n), percentage (%); mean ± standard deviation (SD); minimum–maximum values; and 95% confidence intervals (CIs). The normality of the distribution of continuous variables was assessed using Kolmogorov–Smirnov and Shapiro–Wilk tests, as well as by examining skewness and kurtosis values. Parametric or nonparametric statistical methods were employed depending on the distribution characteristics. For comparisons between two groups, the independent samples t-test was used for normally distributed variables and the Mann–Whitney U test for non-normally distributed variables. For comparisons involving three or more groups, one-way analysis of variance (ANOVA) was used when the normality assumption was met; otherwise, the Kruskal–Wallis test was used. Homogeneity of variances was assessed using Levene’s test. Spearman correlation analysis was used to examine relationships between continuous variables. A *p*-value of less than 0.05 was considered statistically significant for all analyses.

## 3 Results

A total of 76 participants were included in the study: 40 patients diagnosed with bladder cancer and 36 healthy individuals. Participants’ ages ranged from 41 to 82 years, with an average age of 63.04 ± 10.15 years. The mean body mass index (BMI) was 28.31 ± 4.79 kg/m^2^. The demographic characteristics of the groups are summarised in Table 1. There were no significant differences between the patient and control groups in terms of age, height, weight, BMI or smoking history (all *p* > 0.05). Although a noticeable difference in sex distribution was present between the groups, no statistical comparison was performed for this variable. However, the imbalance has been identified as a possible source of bias. All patients in the bladder cancer group were diagnosed with non-muscle-invasive transitional cell carcinoma (urothelial carcinoma). The mean microplastic concentration was 2.82 ± 2.05 particles/ml in the control group and 2.53 ± 1.54 particles/ml in the bladder cancer group. No statistically significant difference was observed between the groups regarding microplastic concentration (*p* = 0.868). The mean microplastic concentration was 2.82 ± 2.05 particles/ml in the control group; 2.45 ± 1.43 particles/ml in the group with low-grade non-muscle-invasive bladder cancer; and 2.64 ± 1.71 particles/ml in the group with high-grade bladder cancer. Group comparisons are presented in Table 2. Within the bladder cancer group, microplastic concentration did not differ significantly according to sex, the presence of lamina propria invasion, tumour grade, tumour size, the number of tumour foci, the presence of carcinoma *in situ* (CIS) or smoking history (all *p* > 0.05). Microplastic concentrations across subgroups of bladder cancer patients are summarised in Table 3. Spearman correlation analysis revealed no significant association between microplastic concentration and age, BMI, or tumour size (all *p* > 0.05).

**Table 1:** Demographic characteristics of the study population.

Variable	Bladder cancer Group (*n* = 40)	Control group (*n* = 36)	Total (*n* = 76)	*p*-value
Age (years) Mean+SD (min–max)	61.33 ± 8.57 (44–80)	59.39 ± 10.62 (41–82)	63.04 ± 10.15 (41–82)	0.112^a^
Height (cm) Mean+SD (min–max)	170.20 ± 8.63 (153–185)	168.72 ± 9.39 (150–181)	169.50 ± 8.97 (150–185)	0.477^a^
Weight (kg) Mean+SD (min–max)	81.25 ± 13.15 (55–120)	80.92 ± 13.78 (48–120)	81.09 ± 13.36 (48–120)	0.914^a^
BMI (kg/m^2^) Mean+SD (min–max)	28.13 ± 4.66 (19.41–40.57)	28.50 ± 4.98 (19.59–40.89)	28.31 ± 4.79 (19.41–40.89)	0.934^a^
Sex (F/M) *n* (%)	5/35 (12/88)	26/10 (72/28)	31/45 (41/59)	**<0.001** ^b^
Smoking history	26 (%65)	—	26 (%34)	—

Categorical variables were compared using the a ^a^Chi-square test, For data showing a normal distribution in comparisons between the two groups,^b^an independent t-test was applied and the min, max, and SD values were given. Min, minimum; max, maximum; SD, standard deviation; BMI, body mass index; F, female; M, male. Bold values indicate statistically significant results (*p* < 0.05).

**Table 2:** Microplastic concentrations across study groups.

Group	Mean ± SD (particles/ml)	Min–Max (particles/ml)	*p* value	
Control group (*n* = 36)	2,82 ± 2,05	0,67 – 9,33		0.868
Low-grade disease (*n* = 22)	2.45 ± 1.43	0.73 – 7.00	0.881	
High-grade disease (*n* = 18)	2.64 ± 1.71	0.67 – 7.00		
Total	2.67 ± 1.79	0.67 – 9.33	—	—

For data showing a normal distribution in comparisons between the two groups, an independentt-test was applied and the min, max, and SD values were given. Min, minimum; max, maximum; SD, standard deviation.

**Table 3:** Microplastic concentration according to clinicopathological variables in bladder cancer patients.

Variable	Category	Mean ± sd (particles/ml)	*p*-value
Sex	Female	2.99 ± 1.65	0.390
	Male	2.47 ± 1.54	
Lamina propria invasion	Absent	2.50 ± 1.60	0.343
	Present	2.85 ± 1.01	
Tumor grade	Low	2.70 ± 1.65	0.498
	High	2.37 ± 1.46	
Number of tumor foci	Multiple	2.52 ± 1.62	0.721
	Single	2.60 ± 1.34	
Presence of CIS	Absent	2.60 ± 1.63	0.786
	Present	2.28 ± 1.22	
Smoking status	Non-smoker	2.51 ± 1.29	0.410
	Smoker	2.27 ± 1.59	
	Former smoker	3.51 ± 1.81	
Tumor size	< 3 cm	2.54 ± 1.36	0.646
	≥ 3 cm	2.82 ± 1.68	

For data showing a normal distribution in comparisons between the two groups, an independent t-test was applied and the min, max, and SD values were given. SD, standard deviation; CIS, carcinoma *in Situ*.

## 4 Discussion

Urothelial carcinoma of the bladder is one of the most common cancers worldwide. Exposure to polycyclic aromatic hydrocarbons, nitrosamines, aromatic amines, aldehydes, phenols, volatile hydrocarbons and heavy metals is a well-established cause of the disease, primarily through inducing base alterations and double-strand DNA breaks that can lead to gene mutations and disruption of tumour suppressor pathways (20, 21). Plastics, which are made up of carbon-based polymers with various chemical additives (many of which are toxic), have emerged as a trending environmental concern. MPs are widely distributed in air and water and are increasingly recognised as environmental contaminants with the potential to affect biology. They can also be detected in human urine (12, 17). However, their potential involvement in bladder carcinogenesis remains unclear. As the bladder is in storage phase for most of the day, with urine in prolonged contact with the urothelium, investigating urinary MPs in the context of bladder cancer is biologically relevant. This is particularly relevant given that the bladder epithelium is exposed to the contents of the bladder for prolonged periods. In this study, MPs were detected in urine samples from both healthy individuals and patients with bladder cancer, which is consistent with previous reports identifying MPs in the urine of healthy volunteers (22). The relatively large sample size, compared with that of previous studies, strengthens the validity of these findings. Our results showed that urinary MP concentrations were similar in bladder cancer patients and the control group. These findings contradict previous reports that suggested MPs could induce inflammation, oxidative stress and DNA damage. These mechanisms have been associated with the development of cardiovascular, respiratory and malignant diseases (23). A recent meta-analysis reviewing case control studies also suggested a weak correlation between MP exposure and cancer risk (24). It has been hypothesised that MPs promote uncontrolled cell proliferation and contribute to malignancies such as lung, haematological, breast, prostate and ovarian cancers (10); however, the underlying mechanisms remain poorly defined. Accumulation of MPs in tumour tissue has been reported in other organ systems. For example, significant MP deposition has been identified in breast cancer tissue (25) and penile cancer specimens using laser infrared (LDIR) spectroscopy (26). MPs have also been implicated in promoting squamous cell carcinoma of the skin by inducing epithelial injury and increasing tumour cell proliferation (27). To date, no study has evaluated the presence of MPs in bladder tumour tissue. In our study, we only examined urine samples; therefore, it remains possible that MPs adhere preferentially to tumour surfaces rather than being excreted into urine. Future research should explore the presence and distribution of MPs within bladder tumour specimens directly. Within the bladder cancer group, urinary MP concentration did not differ according to lamina propria invasion, tumour grade, tumour size, number of tumour foci, CIS status or smoking history. Thus, no association was found between MP load and disease severity or pathological aggressiveness. However, some studies have suggested that MPs may enhance tumour progression by promoting cell migration (14) or altering the tumour microenvironment and impairing immune surveillance (13). These mechanisms imply that MPs could contribute to metastatic processes, even if urinary concentrations remain unchanged. Evidence from colorectal cancer research has suggested that MPs may disrupt mucosal barriers and contribute to sporadic malignancies that occur at an early age (11). A similar mechanism could theoretically damage the glycosaminoglycan layer of the bladder and promote urothelial injury. However, our findings do not support this hypothesis. Furthermore, preclinical models have indicated that MPs may enhance cancer incidence and mediate chemoresistance via the mTOR/ULK1 axis (28), raising concerns about their impact on outcomes in advanced-stage cancer. As our cohort did not include muscle-invasive or metastatic bladder cancer, we cannot draw conclusions regarding advanced disease. The mechanism by which MPs enter the urinary collecting system suggests that smaller particles, particularly nanoplastics, are more likely to be filtered through the glomeruli and excreted in the urine (29, 30). As our analytical approach was unable to detect nanoplastics — smaller particles with a higher degree of biological penetrability — it is possible that clinically relevant differences may exist at the nanoscale level. Nanoplastics are more likely to undergo glomerular filtration and interact directly with urothelial cells, potentially exerting a greater biological effect than larger microplastic particles. Therefore, future studies should incorporate nanoplastic quantification to better clarify their potential role in bladder carcinogenesis. Environmental exposure to chemical contaminants, including heavy metals, is known to contribute to bladder cancer pathogenesis (16). MPs have been proposed to act as ‘Trojan horses’ by absorbing and transporting toxic organic pollutants and heavy metals within the body (31). Thus, their potential role in bladder cancer may extend beyond direct toxicity to include the facilitation of established carcinogens. In addition, this study did not quantitatively assess several environmental and lifestyle-related confounding factors. Factors such as occupational exposure, dietary habits (including bottled water consumption and seafood intake) and regional water quality may significantly impact individual microplastic exposure levels, potentially obscuring subtle associations. The absence of these parameters may have obscured subtle associations between microplastic burden and bladder cancer risk.

Furthermore, previous reports have linked environmental contamination associated with plastic waste to increased regional incidences of breast and bladder cancer (32), and evidence suggests that approximately 10% of annual bladder cancer cases in the United States may be attributable to disinfectant by-products in drinking water (33). This supports the investigation of MPs as potential environmental contributors to carcinogenesis. In our study, urinary MP concentrations were similar between the sexes, despite known sex-specific differences in occupational exposure. This finding is consistent with studies demonstrating the presence of MPs in the airways of indoor and outdoor workers (34), suggesting that exposure through inhalation and water sources may be a more widespread, gender-independent route of exposure. However, this finding should be interpreted with caution due to the imbalance in sex distribution between the study groups.

## 5 Strengths and limitations

This study has several key strengths. Firstly, the relatively large sample size enhances the reliability and robustness of the findings. Secondly, the rigorous, multi-step methodology used for microplastic identification is a significant advantage. Not only were microplastics evaluated based on morphological characteristics, they were also verified using the hot needle test and confirmed with Nile Red fluorescence staining. Integrating these complementary techniques improves detection accuracy and reduces the likelihood of particle misclassification. However, several limitations should be acknowledged. The study was conducted at a single centre with a relatively small sample size, which may restrict the generalisability of the findings. Furthermore, only urine samples were analysed, meaning that potential microplastic accumulation within bladder tumour tissue or normal urothelium was not assessed. Papillary tumour surfaces may retain microplastics more readily than urine, which could lead to an underestimation of the true microplastic burden in patients with bladder cancer. Another important limitation is the lack of spectroscopic confirmation techniques such as Fourier-transform infrared (FTIR) or Raman spectroscopy, which are considered the gold standard for polymer identification. Although Nile Red staining is widely used, it may also bind to non-plastic organic materials, such as lipids, which introduces a risk of false-positive identification. Additionally, due to technical and methodological constraints, the polymer types of the detected microplastics (e.g., polyethylene, polystyrene, polyurethane and PVC) could not be determined. Furthermore, the analytical methods used in this study were incapable of detecting nanoplastics, which may be more biologically relevant due to their ability to penetrate tissues and interact at the cellular level. In addition, some control subjects presented with microscopic hematuria, which may have influenced urinary particulate content and potentially affected microplastic quantification. Although no malignancy was detected on cystoscopy and during follow-up, the possibility of missed flat lesions such as carcinoma *in situ* cannot be completely excluded. Using a single spot urine sample is another limitation as this may not accurately reflect long-term cumulative exposure to microplastics. Longitudinal sampling approaches would provide a more reliable assessment. Finally, environmental and individual exposure factors, including occupational risks, dietary habits and local water sources, were not quantitatively evaluated and may have influenced microplastic exposure levels at an individual level. Additionally, the imbalance in sex distribution between the study groups may represent a potential source of bias and should be considered when interpreting the results.

## 6 Conclusion

Our literature review revealed that no prior studies had directly compared microplastic concentrations in urine with the presence of human bladder cancer. Within the methodological constraints of the present study, no statistically significant association was observed between urinary microplastic concentration and bladder cancer. Furthermore, microplastic concentration did not predict any of the clinical or pathological parameters examined among patients with bladder cancer. In light of the increasing global exposure to environmental plastics, these results emphasise the necessity for further research into plastic-related carcinogenesis. Further research is required in the form of larger cohort studies with broader sample sizes to better elucidate the potential relationship between microplastic concentration in urine and the development of bladder cancer.

## Data Availability

The original contributions presented in the study are included in the article/supplementary material, further inquiries can be directed to the corresponding author.
